# Comparison of *Leishmania* typing results obtained from 16 European clinical laboratories in 2014

**DOI:** 10.2807/1560-7917.ES.2016.21.49.30418

**Published:** 2016-12-08

**Authors:** Gert Van der Auwera, Aldert Bart, Carmen Chicharro, Sofia Cortes, Leigh Davidsson, Trentina Di Muccio, Jean-Claude Dujardin, Ingrid Felger, Maria Grazia Paglia, Felix Grimm, Gundel Harms, Charles L. Jaffe, Monika Manser, Christophe Ravel, Florence Robert-Gangneux, Jeroen Roelfsema, Seray Töz, Jaco J. Verweij, Peter L. Chiodini

**Affiliations:** 1 Biomedical Sciences, Institute of Tropical Medicine, Antwerp, Belgium; 2 Academic Medical Center, Amsterdam, The Netherlands; 3 Instituto de Salud Carlos III, Madrid, Spain; 4 Global Health and Tropical Medicine, GHTM, Instituto de Higiene e Medicina Tropical, UNL, Lisbon, Portugal; 5 The Public Health Agency of Sweden, Stockholm, Sweden; 6 Istituto Superiore di Sanità, Rome, Italy; 7 Biomedical Sciences, Antwerp University, Antwerp, Belgium; 8 Swiss Tropical and Public Health Institute, Basel, Switzerland; 9 University of Basel, Basel, Switzerland; 10 National Institute for Infectious Diseases (INMI) Lazzaro Spallanzani, Rome, Italy; 11 Institute of Parasitology, University of Zürich, Zürich, Switzerland; 12 Institute of Tropical Medicine and International Health, Charité-Universitätsmedizin Berlin, Berlin, Germany; 13 Hebrew University, Hadassah Medical Centre, Jerusalem, Israel; 14 United Kingdom National External Quality Assessment Service, London, United Kingdom; 15 University of Montpellier, Montpellier, France; 16 Centre Hospitalier Universitaire de Rennes, Rennes, France; 17 National Institute for Public Health and the Environment, RIVM, Bilthoven, The Netherlands; 18 Ege University, Faculty of Medicine, Department of Parasitology, Izmir, Turkey; 19 St. Elisabeth Hospital, Tilburg, The Netherlands; 20 Hospital for Tropical Diseases, London, United Kingdom; 21 London School of Hygiene and Tropical Medicine, London, United Kingdom

**Keywords:** leishmaniasis, Typing, hsp70, rDNA ITS1, mini-exon, kinetoplast DNA

## Abstract

Leishmaniasis is endemic in southern Europe, and in other European countries cases are diagnosed in travellers who have visited affected areas both within the continent and beyond. Prompt and accurate diagnosis poses a challenge in clinical practice in Europe. Different methods exist for identification of the infecting *Leishmania* species. Sixteen clinical laboratories in 10 European countries, plus Israel and Turkey, conducted a study to assess their genotyping performance. DNA from 21 promastigote cultures of 13 species was analysed blindly by the routinely used typing method. Five different molecular targets were used, which were analysed with PCR-based methods. Different levels of identification were achieved, and either the *Leishmania* subgenus, species complex, or actual species were reported. The overall error rate of strains placed in the wrong complex or species was 8.5%. Various reasons for incorrect typing were identified. The study shows there is considerable room for improvement and standardisation of *Leishmania* typing. The use of well validated standard operating procedures is recommended, covering testing, interpretation, and reporting guidelines. Application of the internal transcribed spacer 1 of the rDNA array should be restricted to Old World samples, while the heat-shock protein 70 gene and the mini-exon can be applied globally.

## Introduction

Leishmaniasis is a vector-borne disease which is endemic in 98 countries worldwide [1]. It is caused by protozoan parasites of the genus *Leishmania*, which are transmitted by female sand flies of the genera *Lutzomyia* and *Phlebotomus*. Many infected individuals never develop symptoms, but those who do can exhibit various disease manifestations [2]. Visceral leishmaniasis (VL) or kala-azar is the severe form, whereby parasites infect internal organs and the bone marrow, a lethal condition if left untreated. Other disease types are restricted to the skin (cutaneous leishmaniasis, CL) or the mucosae of the nose and mouth (mucosal leishmaniasis, ML). Finally, a particular cutaneous disease sometimes develops in cured VL patients: post kala-azar dermal leishmaniasis (PKDL). Typically, VL is caused by two species: *Leishmania donovani* and *Leishmania infantum*. The latter can also cause CL, as can all other pathogenic species. Some particular species (e.g. *L. braziliensis* and *L. aethiopica*) can lead to overt ML.

As many as 20 different *Leishmania* species are able to infect humans, and globally there are over 1 million new disease cases per annum [1,3]. Leishmaniasis is endemic in southern Europe, and in other European countries cases are diagnosed in travellers who have visited affected areas both within the continent and beyond. Although treatment in practice is often guided only by clinical presentation and patient history, in some cases determination of the aetiological subgenus, species complex or species is recommended for providing optimal treatment [2,4,5]. For example, a patient returning from South America with CL might be infected with *Leishmania braziliensis*, which necessitates systemic drug therapy and counselling about the risk of developing mucosal leishmaniasis in the future. The same patient could also be infected with *Leishmania mexicana*, which is managed by less intensive treatment and which is not associated with mucosal disease [6]. Determining the infecting species and its probable source permits selection of the correct drug, route of administration (intralesional, oral systemic, or parenteral) and duration [7].

Unfortunately, for CL it is impossible to predict the species responsible for an ulcerating lesion clinically, and the morphology of amastigotes does not differ between species. When the geographical origin of infection is known, for instance when a patient in an endemic region is treated at a local hospital, the species can be guessed often from the known local epidemiology, as species distribution follows a geographical pattern [8]. However, especially in infectious disease clinics that treat patients who have stayed in various endemic countries, the geographic origin of infections may be unknown. For instance, people residing in Europe who have travelled outside Europe may come from, or have also visited, *Leishmania*-endemic areas within Europe, especially the Mediterranean basin. Even when the location of infection is known, several species can co-circulate in a given endemic area, in which case the species can only be determined by laboratory tests. Culture and subsequent isoenzyme analysis is time consuming and available in very few specialised centres, so it is impractical as a front-line diagnostic test in clinical laboratories. Hence, well-performed reliable molecular methods are necessary for species identification.

Several *Leishmania* typing methods have been published (reviewed in [9]), and as a result each laboratory uses its own preferred assay. The most popular assays nowadays are those that can be applied directly to clinical samples, thereby circumventing the need for parasite isolation and culture. However, few tests have been standardised, and no commercial kits are currently available. As a result, clinical and epidemiological studies make use of various techniques, and in patient management other methods are often deployed. In this study we compare the typing performance in 16 clinical laboratories across Europe, which use a variety of methods for species discrimination.

## Methods

### Participants and reference methods

Twenty one *Leishmania* isolates were typed by 16 laboratories in 12 countries in 2014. Table 1 lists the parasite strains that were used in this study, along with the reference method for species identification. Strains identified with a Laboratoire d'Ecologie Médicale (LEM) code were provided by the Centre National de Référence des Leishmanioses in Montpellier, France, which assigns LEM codes to each cryopreserved culture, while the remaining three strains were provided by the Institute of Tropical Medicine in Antwerp, Belgium.

**Table 1:** Strains used, study comparing *Leishmania* typing results in 16 European clinical laboratories, 2014

Strain (WHO code)	Culture name CNRL^a^	Species^b^	Reference typing method^c^
MHOM/ET/83/130–83	LEM1118	*Leishmania aethiopica*	MLEE, MLSA
MHOM/GF/2002/LAV003	LEM4351	*L. amazonensis*	MLEE, MLSA
MHOM/VE/76/JAP78	LEM0391	*L. amazonensis*	MLEE, MLSA
MHOM/BR/75/M2903b	LEM0396	*L. braziliensis*	MLEE, MLSA
MHOM/PE/83/STI139	LEM0781	*L. braziliensis*	MLEE, MLSA
MHOM/BO/2001/CUM555	NA	*L. braziliensis* outlier^d^	AFLP [12], WGS, MLSA
MHOM/IN/--/LRC-L51	LEM1070	*L. donovani*	MLEE, MLSA
MHOM/KE/55/LRC-L53	LEM0707	*L. donovani*	MLEE, MLSA
MHOM/GF/86/LEM1034	LEM1034	*L. guyanensis*	MLEE, MLSA
MHOM/FR/78/LEM75	LEM0075	*L. infantum*	MLEE, MLSA
MCUN/BR/85/M9342	LEM2229	*L. lainsoni*	MLEE, MLSA
MHOM/IQ/86/CRE1	LEM0858	*L. major*	MLEE, MLSA
MHOM/BZ/82/BEL21	LEM0695	*L. mexicana*	MLEE, MLSA
MHOM/EC/87/EC103-CL8	LEM1554	*L. mexicana*	MLEE, MLSA
MDAS/BR/79/M5533	LEM2204	*L. naiffi*	MLEE, MLSA
MHOM/CO/86/UA126	LEM1047	*L. panamensis*	MLEE, MLSA
MHOM/CO/88/UA264	LEM1492	*L. panamensis*	MLEE, MLSA
MHOM/CO/88/UA316	LEM1505	*L. panamensis* / *L. guyanensis*^e^	MLEE, MLSA
MHOM/PE/90/HB86	NA	*L. peruviana*	AFLP [12], WGS, MLSA
MHOM/PE/90/LCA08	NA	*L. peruviana*	AFLP [12], WGS, MLSA
MHOM/IL/80/SINGER	LEM0617	*L. tropica*	MLEE, MLSA

AFLP: amplified fragment length polymorphism; CNRL: Centre National de Référence des Leishmanioses (Montpellier, France); NA: not applicable; MLEE: multilocus enzyme electrophoresis; MLSA: multilocus sequence analysis; WGS: whole genome sequencing; WHO: World Health Organization.

^a^ Identification in the Montpellier cryobank (Centre National de Référence des Leishmanioses).

^b^ For the taxonomic position of each species (subgenus and species complex), please refer to Figure 2.

^c^ Reference method used to determine the species of each isolate. MLEE [10]; MLSA based on seven genes [11]; AFLP analysis [12]; WGS (unpublished results).

^d^ Group of distinct *Leishmania braziliensis* strains [9,12], also called *L. braziliensis* type 2 [15] or atypical *L. braziliensis* [18].

^e^ This strain was typed as *L. panamensis* by MLSA, and as *L. guyanensis* by MLEE.

Four highly informative reference methods were used: multilocus enzyme electrophoresis (MLEE [10]), multilocus sequence analysis (MLSA [11], GenBank sequence accession numbers in Table 2), genome-wide amplified fragment length polymorphism (AFLP) analysis [12], and whole genome sequencing (unpublished results).

**Table 2:** GenBank sequence accession numbers from MLSA and *hsp70,* for sequences used in study comparing *Leishmania* typing results in 16 European clinical laboratories, 2014

WHO CODE	LEM	MLSA locus							*hsp70*
		3,0980	4,0580	10,0560	12,0010	14,0130	31,0280	31,2610	
MCUN/BR/85/M9342	2229	KT959002	KT959017	KT959032	KT959047	KT959062	KT959077	KT959092	LN907839
MDAS/BR/79/M5533	2204	KT959001	KT959016	KT959031	KT959046	KT959061	KT959076	KT959091	FR872767
MHOM/BO/2001/CUM555	NA	KT959006	KT959021	KT959036	KT959051	KT959066	KT959081	KT959096	FR872760
MHOM/BR/75/M2903b	396	KT958993	KT959008	KT959023	KT959038	KT959053	KT959068	KT959083	LN907832
MHOM/BZ/82/BEL21	695	KT958994	KT959009	KT959024	KT959039	KT959054	KT959069	KT959084	LN907841
MHOM/CO/86/UA126	1047	KT958997	KT959012	KT959027	KT959042	KT959057	KT959072	KT959087	LN907843
MHOM/CO/88/UA264	1492	KT958998	KT959013	KT959028	KT959043	KT959058	KT959073	KT959088	LN907844
MHOM/CO/88/UA316	1505	KT958999	KT959014	KT959029	KT959044	KT959059	KT959074	KT959089	LN907837
MHOM/EC/87/EC103-CL8	1554	KT959000	KT959015	KT959030	KT959045	KT959060	KT959075	KT959090	LN907842
MHOM/ET/83/130–83	1118	KC159315	KC159537	KC159093	KC159759	KC158871	KC159981	KC158649	LN907830
MHOM/FR/78/LEM75	75	KC159255	KC159477	KC159033	KC159699	KC158811	KC159921	KC158589	LN907838
MHOM/GF/2002/LAV003	4351	KT959003	KT959018	KT959033	KT959048	KT959063	KT959078	KT959093	LN907831
MHOM/GF/86/LEM1034	1034	KT958996	KT959011	KT959026	KT959041	KT959056	KT959071	KT959086	LN907836
MHOM/IL/80/SINGER	617	KC159287	KC159509	KC159065	KC159731	KC158843	KC159953	KC158621	LN907846
MHOM/IN/--/LRC-L51	1070	KC159313	KC159535	KC159091	KC159757	KC158869	KC159979	KC158647	LN907834
MHOM/IQ/86/CRE1	858	KC159299	KC159521	KC159077	KC159743	KC158855	KC159965	KC158633	LN907840
MHOM/KE/55/LRC-L53	707	KC159294	KC159516	KC159072	KC159738	KC158850	KC159960	KC158628	LN907835
MHOM/PE/1990/HB86	NA	KT959004	KT959019	KT959034	KT959049	KT959064	KT959079	KT959094	LN907845
MHOM/PE/1990/LCA08	NA	KT959005	KT959020	KT959035	KT959050	KT959065	KT959080	KT959095	EU599089
MHOM/PE/83/STI139	781	KT958995	KT959010	KT959025	KT959040	KT959055	KT959070	KT959085	LN907833
MHOM/VE/76/JAP78	391	KT958992	KT959007	KT959022	KT959037	KT959052	KT959067	KT959082	EU599092

LEM: Laboratoire d'Ecologie Médicale; MLSA: multilocus sequence analysis; *hsp70*: heat-shock protein 70 gene; NA: not applicable; WHO: World Health Organization.

DNA was extracted from parasite cultures using either the DNeasy Blood and Tissue Kit or QIAamp DNA Mini Kit (Qiagen, www.qiagen.com), and the concentration was measured spectrophotometrically. The 21 DNAs were randomised at the United Kingdom (UK) National External Quality Assessment Service for Parasitology (UKNEQAS, London, UK), and every study participant received a blind panel containing 50 µl of a 10 ng/µl DNA solution. The participating laboratories are listed in Table 3.

**Table 3:** Participants in study comparing *Leishmania* typing results in 16 European clinical laboratories, 2014

Institute	City	Country
Institute of Tropical Medicine Antwerp^a^	Antwerp	Belgium
Centre National de Référence des Leishmanioses^a,b^	Montpellier	France
Centre Hospitalier Universitaire de Rennes	Rennes	France
Charité-Universitätsmedizin Berlin	Berlin	Germany
Hebrew University-Hadassah Medical Centre	Jerusalem	Israel
Istituto Superiore di Sanità	Rome	Italy
National Institute for Infectious Diseases L. Spallanzani	Rome	Italy
Instituto de Higiene e Medicina Tropical	Lisbon	Portugal
Instituto de Salud Carlos III	Majadahonda	Spain
The Public Health Agency of Sweden	Stockholm	Sweden
Swiss Tropical and Public Health Institute^a^	Basel	Switzerland
Institute of Parasitology	Zürich	Switzerland
Academic Medical Center, University of Amsterdam	Amsterdam	The Netherlands
National Institute for Public Health and the Environment	Bilthoven	The Netherlands
St. Elisabeth Hospital	Tilburg	The Netherlands
Ege University Medical School	Izmir	Turkey
Hospital for Tropical Diseases	London	United Kingdom

Institutes are listed in alphabetical order based on country and city.

^a^ These laboratories provided parasite cultures and DNA.

^b^ This laboratory applied one of the reference methods (MLSA) and did not participate in the comparative study of typing outcomes.

After performing the respective routine typing technology, each laboratory reported its results to UKNEQAS, who forwarded these along with the randomised code in one batch to the Institute of Tropical Medicine in Antwerp for analysis. Some participants used the term ‘*L. braziliensis* complex’ when referring to the *L.* (*Viannia*) subgenus, and where needed the reported results were adjusted. The results after these adjustments are presented in this analysis.

### Genome targets for typing

**Figure 1:**
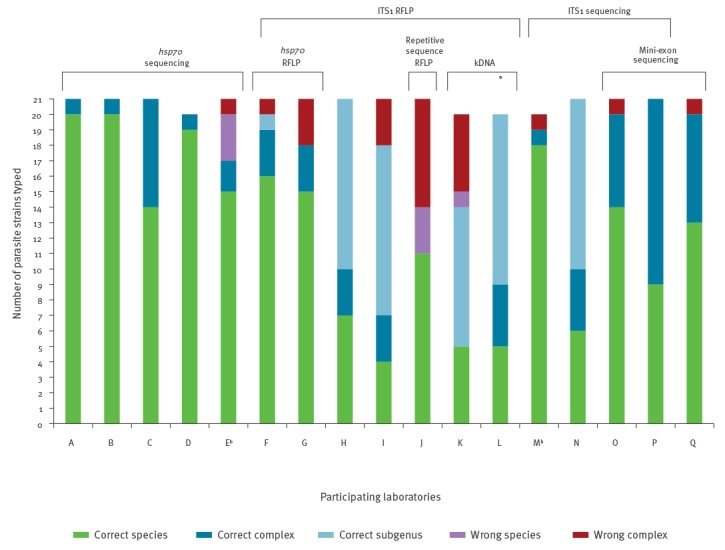
Typing results obtained in study comparing *Leishmania* typing results in 16 European clinical laboratories, 2014

ITS: internal transcribed spacer; *hsp70*: heat-shock protein 70 gene; kDNA: kinetoplast minicircle DNA; RFLP: restriction fragment length polymorphism.

^a^ RFLP was performed on a fragment covering both ITS1 and ITS2 [14].

^b^ One laboratory reported the use of two separate methods. Results E and M.

The 16 laboratories used a total of five genome targets for typing (Table 4): the internal transcribed spacer 1 of the rDNA array (ITS1), the mini-exon, kinetoplast minicircle DNA (kDNA), the heat-shock protein 70 gene (*hsp70*), and a repetitive DNA sequence. One laboratory reported two sets of result from two different targets, which are treated in the analysis as if they were from separate laboratories, which is why the results section describes 17 instead of 16 outcomes. The targets were analysed with PCR, generally followed by sequencing or restriction fragment length polymorphism (RFLP) analysis, as shown in Table 4 and Figure 1. Four laboratories used in-house sequencing, while five others used the service of an external sequencing facility. PCRs based on kDNA did not require post-PCR manipulations other than gel analysis.

**Table 4:** Typing methods used in study comparing *Leishmania* typing results in 16 European clinical laboratories, 2014

Genomic locus / gene	Analysis method	Number of laboratories^a^
ITS1	RFLP [13,14]	7
	Sequencing [15]	4
*hsp70*	Sequencing [15,16]	5
	RFLP [17]	2
Mini-exon	Sequencing [19-21]	3
kDNA minicircles	RFLP [24,25]	1
	Specific PCR [23]	1
Repetitive DNA	RFLP [26]	1

ITS: internal transcribed spacer; *hsp70*: heat-shock protein 70 gene; kDNA: kinetoplast minicircle DNA; RFLP: restriction fragment length polymorphism.

^a^ The total number is higher than the 16 participating laboratories, because several laboratories used different methods in parallel.

Figure 1 indicates for each laboratory individually which method or methods were used, but not all samples were necessarily analysed with each method. Of the 16 laboratories, 11 used the ITS1 target, either applying RFLP (n=7) or sequencing (n=4). All of them based their analysis on the fragment described in [13], except for laboratory L which used a larger region also including ITS2 [14]. Five laboratories based typing on *hsp70*: four (A-D) used sequencing of the F fragment described in [15-17], while one (E) used the N fragment. Two laboratories (F and G) analysed this gene with RFLP [17,18]. Three laboratories used sequence analysis of the mini-exon gene: laboratory O [19,20], laboratory P [21], and laboratory Q [22]. Two laboratories based typing partly on kDNA: laboratory K [23], and laboratory L [24,25]. Finally, laboratory J complemented ITS1-RFLP with RFLP analysis of a repetitive DNA sequence [26].

### Grading of results

**Figure 2:**
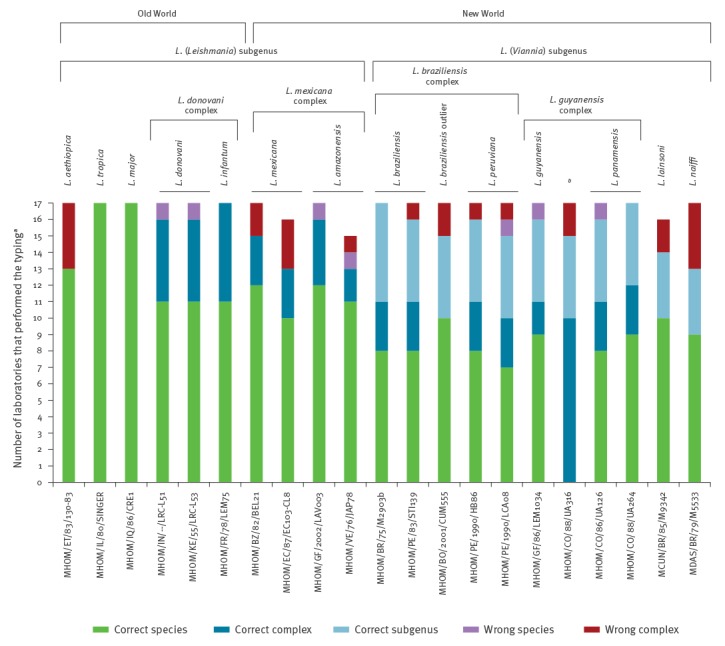
Typing results for each of the 21 strains included in study comparing *Leishmania* typing results in 16 European clinical laboratories, 2014

MLSA: multilocus sequence analysis; WHO: World Health Organization.

^a^ One laboratory reported the use of two separate methods.

^b^ Strain MHOM/CO/88/UA316 is *L. guyanensis* based on MLEE, but *L. panamensis* based on MLSA (Table 1).

Each individual result was graded as follows. The best ranking was given to reported species agreeing with the reference methods, whereby *L. garnhami* was considered a synonym of *L. amazonensis* [27]. Results reporting MHOM/BO/2001/CUM555 as *L. braziliensis* were considered correct. Although this strain belongs to a group of clearly distinguishable outliers (Table 1), it has so far not been described as a separate species. Next were identifications that reported the species complex rather than the actual species (see Figure 2), and were in agreement with the reference methods. The lowest ranking of correct results was given to those identifying the subgenus, i.e. *L.* (*Viannia*) or *L.* (*Leishmania*), without specification of species or species complex. Identification errors were graded at two levels. First, some laboratories reported a species within the correct complex, but identified the wrong species within that complex. Second, some isolates were placed in an erroneous species complex altogether. A peculiar case was presented by strain MHOM/CO/88/UA316, which was *L. guyanensis* based on MLEE, but *L. panamensis* based on MLSA (Table 1). For this strain, all results reporting either *L. guyanensis* or *L. panamensis* were considered to have identified the correct species complex.

In a next level of the analysis, the cause of erroneous typings was sought by means of in-depth assessment of the methods. The reasons for different identification outcomes of laboratories using the same methods were also identified. Sequences from laboratories that based their typing on the same genes were compared by alignment in the software package MEGA5 [28].

## Results

Results from all analyses are summarised in Figure 1, details are available from [29]. One laboratory reported two sets of results because identification based on *hsp70* sequences was sometimes in conflict with those of ITS1 sequencing. These results are listed separately from laboratories E and M respectively, which brings the number of reported result sets from the 16 laboratories to 17. Most laboratories succeeded in typing all 21 samples, but in some cases results were reported for 20/21 isolates only (laboratories D, K, L, M). The total number of erroneous identifications amounted to 30, with 23 of these being classified in an incorrect species complex. On a total of 353 results, these represent 8.5% and 6.5% respectively. The correct species was identified in 211 typing results (60%), while 58 (16%) identified the correct species complex, and 54 (15%) the correct subgenus. Eight laboratories made no incorrect assignments, while the laboratory with most errors (laboratory J) misidentified 10 out of 21 samples, seven of which were placed in the wrong species complex. Laboratories relying only on kDNA and ITS1 more frequently reported results to the subgenus level, while laboratories using the mini-exon or *hsp70* often succeeded in obtaining identification either to the species or complex level.

For each method, the number of correct typings to species, species complex, and subgenus level are shown in different colours. In addition, the incorrect species designations are indicated, some of which identified the wrong species in the correct complex (purple bars), others placing a strain in the wrong complex (red bars). The methods or combination of methods that were used to obtain the given results are shown on top.

Figure 2 depicts the typing results for each strain, irrespective of the methods used. The only two species that were correctly identified with all methods were *L. tropica* and *L. major*. Strains from the *L.* (*Leishmania*) subgenus were identified to either the species or complex level by all laboratories. This was in contrast to the 11 strains from the *L.* (*Viannia*) subgenus, each of which was typed by four to six laboratories only to the subgenus level. The error rate for both subgenera was comparable: 8.4% (14/167) for *L.* (*Leishmania*) and 8.6% (16/186) for *L.* (*Viannia*). The error rate in Old World strains was lower than for strains of the New World: 5.9% (6/102) and 9.6% (24/251) respectively.

For each strain, the number of correct typings at species, species complex, and subgenus level are reported. In addition, the incorrect species designations are indicated, some of which identified the wrong species in the correct complex (purple bars), others placing an isolate in the wrong complex (red bars). The strain identification by WHO code (Table 1) is given with the abscissa. Species, complexes, and subgenera are represented on top, with an indication of the New or Old World strain origin.

When comparing the *hsp70* sequences provided by four laboratories (A-D), there were marked differences in sequence quality. Three laboratories (A, B, C) succeeded in sequencing the entire or nearly entire fragment F [17], with few or no sequence ambiguities. The sequence sets of two laboratories (A and C) contained one insertion and one deletion relative to the other data, indicating sequence mistakes as the gene shows no size variation [15,16]. In contrast, the quality of the fragment F sequences from one laboratory (D) was considerably lower. Sequences were largely incomplete at their 5’ end and to a lesser extent at their 3’ terminus, and numerous insertions, deletions, and unresolved nucleotides (nt) were present. One laboratory (E) sequenced only the N fragment [17], but base calling quality was poor in the 40 terminal 3’ nt. The consensus *hsp70* sequences were deposited in GenBank (Table 2).

Three laboratories (M, N, O) determined the ITS1 sequence of all isolates, while one laboratory (P) sequenced only MHOM/GF/2002/LAV003. The sequences of two laboratories (N and P) covered the entire amplified PCR product, while some of two others (O and M) were incomplete at the termini. Apart from some insertions in the sequences of one laboratory (N) and occasional unresolved nt in those of another (O), the sequences were identical, except for isolate MHOM/CO/88/UA316. Here, up to 9 nt differences were present in a 120 nt stretch.

Three laboratories (O, P, Q) determined the mini-exon sequences. For some strains the sequences of these laboratories were nearly identical, but for others large size differences of the determined fragment were seen, and deletions and nt identity discrepancies were observed. Also, many nt were not fully resolved.

## Discussion

As a general observation, eight laboratories who participated in this comparison typing performance made no errors, and often laboratories using the same typing marker reported different results (Figure 1). Two of the ‘error-free’ laboratories obtained the highest typing accuracy, with 20 out of 21 strains typed to the species level, and strain MHOM/CO/88/UA316 at the complex level. Using our reference methods MLSA and MLEE (Table 1), the latter species could not be classified unequivocally, and hence results placing it in the *L. guyanensis* complex were regarded as correct. These two laboratories (A and B) based their typing on *hsp70* gene sequencing, which was identified as one of the typing methods with the highest resolution in other comparative studies [9,15]. One other laboratory (C) also made use of this method, but typed several strains only to the complex level. Even though the *hsp70* gene often permits distinction between closely related species, separating them is not always straight-forward. For instance, some MLEE-defined *L. guyanensis* have the same sequence as *L. panamensis* [16]. Because identifying the exact species within a given complex can therefore be difficult, one laboratory (C) decided to identify the species complex rather than the exact species in case of doubt. Apparently the low sequence quality obtained by one of the participants (D) had no adverse effects on the results, probably because species-specific nt identities were not affected. The sequence quality was not influenced by the use of in-house vs external sequencing services.

One laboratory (E) reported four mistakes based on *hsp70* sequences. As opposed to laboratories A-D, the analysis was based on a smaller part of the gene, fragment N [17], which is not suited for typing all species [15]. Nevertheless, several of these species were called based on a BLAST search in GenBank [https://blast.ncbi.nlm.nih.gov/Blast.cgi?PAGE_TYPE=BlastSearch], from which the first listed species was regarded as the final result, regardless of identical similarity scores obtained from other species. In this process some species were by chance determined correctly, while others were erroneously identified. This stresses the importance of correctly interpreting output lists generated by BLAST, because different species can have the same similarity score when the marker is too conservative for discriminating between them. To avoid such errors the species complex rather than the species itself should have been reported. On one occasion, the applied methodology even identified an erroneous complex, i.e. MHOM/ET/83/130–83 was typed as *L. donovani* instead of *L. aethiopica*, based on an erroneous annotation in GenBank. Indeed, several GenBank entries of [30] were wrongfully submitted as *L. donovani*, while they derived in fact from other species [16]. This illustrates the importance of critically evaluating BLAST results, and underscores the importance of an agreed reference panel of sequences from trustworthy laboratories and knowledge of the limitations of a typing marker.

The same laboratory E reported a second results set based on ITS1 sequence analysis, listed under laboratory M in Figure 1. Again, BLAST analysis was applied, and even though ITS1 is not suitable for discriminating *L. braziliensis* and *L. guyanensis* complex species [15], several species were reported. Except for one misclassified *L. braziliensis* outlier strain (Figure 2), species were correctly assigned by laboratory M. However, in several cases also other species showed the same similarity scores, and hence there was no ground for naming the exact species. In contrast, another laboratory (N), which also used ITS1 sequence analysis, reported *L.* (*Viannia*) strains at subgenus level with no further attempt to determine the complex or species. Thereby they respected the limitations of ITS1, although some *L.* (*Viannia*) complexes could have been identified based on their data.

The majority of study participants that used ITS1 did not sequence the target, but relied on RFLP analysis. Laboratories basing their results on this method reported some typical errors: *L. tropica* was mixed up with *L. aethiopica*; the *L. donovani* complex was confused with *L. mexicana*; unsuccessful attempts were made to separate *L. infantum* from *L. donovani*; and on one occasion *L. amazonensis* was identified as *L. major*. When digesting the PCR products with the popular enzyme *Hae*III, sufficient gel resolution is needed in order not to mix up the aforementioned species, as their RFLP fragments are similar in size. In addition, contrary to what was originally published [13], *L. infantum* cannot be distinguished from *L. donovani* [9] and therefore ITS1 can only type to the *L. donovani* complex, without further specification.

Two laboratories (F and G) complemented ITS-RFLP with *hsp70*-RFLP, and both mistook *L. naiffi* for *L. braziliensis*. This is a result of identical patterns generated from *L. naiffi* and many *L. braziliensis* strains with restriction endonucleases *Hae*III and *Rsa*I. The mistake could have been avoided by using the appropriate enzyme *Sdu*I [18].

Only one laboratory (J) made use of a repetitive DNA sequence originally described in [31]. In combination with ITS1, 10 out of the 21 typings were incorrect, whereby seven strains were assigned to the wrong complex. Of the 10 mistakes, nine were made in the *L.* (*Viannia*) subgenus, while the remaining error was due to the unsuccessful separation of *L. infantum* from *L. donovani*. ITS1-RFLP is not suitable for discriminating these species, and the repetitive sequence RFLP was designed for typing Old World strains, where only the *L.* (*Leishmania*) subgenus is encountered. Such mistakes once more underline the importance of knowing the limits of the typing marker chosen.

Kinetoplast DNA is primarily a useful marker to discriminate the two *Leishmania* subgenera, but is less suited for typing to the actual species level (reviewed in [9]). In combination with the fact that also ITS1-RFLP does not discriminate many *L.* (*Viannia*) species, the two laboratories (K and L) using these methods reported typing mostly to the subgenus or species complex level. One of them (K) had a particularly high error rate (6/20) using these markers, probably related to the previously mentioned gel resolution problems and separation of *L. infantum* from *L. donovani* with ITS1-RFLP. In addition the laboratory used ‘*L. braziliensis* complex / *L. guyanensis* complex’ as a synonym for *L.* (*Viannia*), while two strains were *L. naiffi* and *L. lainsoni*.

With the mini-exon sequences, only two mistakes were reported. One laboratory (O) identified *L. mexicana* strain MHOM/EC/87/EC103-CL8 as *L. donovani*, but after disclosing the results realised a mistake in reporting, as their analysis actually did show the correct species. In a comparative analysis of four markers [15], the mini-exon together with *hsp70* were identified as the most discriminative markers worldwide, which is confirmed by the results presented here. Some species within the complexes can, however, not be resolved based on the mini-exon, as also reflected in the current analysis, where often complexes rather than species were identified.

When looking at the typing results for each of the 21 strains (Figure 2), it is apparent that strains of the *L.* (*Viannia*) subgenus were more often typed to the subgenus level, while those of the *L.* (*Leishmania*) subgenus were more often reported at the species level. Given that ITS1 was the most popular marker, this is a logical result in view of the poor discrimination of *L.* (*Viannia*) species by ITS1. Also the fact that for Old World strains 5.9% of typings were erroneous, in comparison to 9.6% New World strains, relates to the use of methods that are tailored to Old World strains. Only two strains were identified to the species level by all laboratories and all methods: MHOM/IL/80/SINGER (*L. tropica*) and MHOM/IQ/86/CRE1 (*L. major*). The results show that several laboratories are currently unable to discriminate *L.* (*Viannia*) species, which is partly explained by the participation in the study of six groups that are situated in a European country where *Leishmania* is actively transmitted. Hence, they mainly diagnose patients infected by endemic species, and use methods primarily tailored to species in the Old World. On the contrary, the remaining laboratories are dealing only with imported leishmaniasis cases, which can originate from anywhere in the world, and for which the origin of infection is sometimes unknown. This forces them to apply assays that are able to identify species from everywhere around the globe.

With regard to nomenclature, there is an evident need for standardisation. When the first results were reported, several laboratories used the term ‘*L. braziliensis* complex’ to refer to *L.* (*Viannia*). For many years these have been synonyms, but current literature restricts this term to *L. braziliensis* and *L. peruviana* [27]. Another confusion can arise from the fact that each complex bears the name of one of its constituent species. For instance, a typing outcome reported as ‘*L. guyanensis*’ has to be clearly distinguished from ‘*L. guyanensis* complex’. Even though this particular problem did not seem to occur in our analysis, one could easily envision such occurrence. One laboratory (K) reported several results as ‘*L. braziliensis* complex / *L. guyanensis* complex’ for referring to *L.* (*Viannia*), but with this term *L. naiffi* and *L. lainsoni* were excluded.

Finally, the particular case of strain MHOM/CO/88/UA316 draws attention to problems in species definitions, as this strain was typed as *L. guyanensis* with MLEE, but as *L. panamensis* with MLSA (Table 1). Reported correct results for this strain were either *L. guyanensis* complex, *L. guyanensis*, or *L. panamensis*, but this was irrespective of the method or target used [29]. Such occasional dubious results are unavoidable when dealing with closely related species, in particular *L. guyanensis-L. panamensis*; *L. braziliensis-L. peruviana*; *L. mexicana-L. amazonensis*; and *L. donovani-L. infantum* [9]. Also newly documented parasite species such as *L. martiniquensis* [32] and *L. waltoni* [33], and variants as the *L. braziliensis* outlier [9,12,15,18] further complicate the interpretation of typing results. It is therefore of utmost importance that species identification is performed with a well-documented standard operating procedure (SOP), clearly describing not only experimental procedures, but also in detail how results should be analysed, interpreted, and reported.

The current study was performed on cultured parasite isolates, so all participants received a high amount of pure parasite DNA. Yet, 8.5% errors were seen, and in four cases no result was obtained. When dealing with patient material, the amount of parasite DNA is much lower, and vastly exceeded by human DNA. As the current study did not assess the sensitivity of the methods used, it is expected that typing success based on clinical samples will be considerably lower. In view of the fact that only recognised reference laboratories participated in this study, there is a clear need for optimisation. On the other hand, in many clinical settings the suspected origin of infection can help in interpretation of typing outcomes, thereby possibly lowering the error rate.

## Conclusions

There is considerable room for improvement of current *Leishmania* typing strategies, and inter-laboratory comparisons such as the one we conducted can contribute to enhance typing quality. Whichever the clinical need for determining the subgenus, complex, or species, and whichever the technology used in a particular setting, typing should be based on a well-defined and validated SOP designed by an expert in *Leishmania* taxonomy. This SOP should cover not only testing, but also analysis and interpretation procedures, and a clear description of how species should be named and reported, taking into account the limitations of each marker and technique, and the problem of resolving closely related species or occasional inter-species hybrids. Validation should be performed on a sufficient amount of reference isolates from various geographic origins to cover each species’ variability. When using sequencing, sequence errors should be avoided, and a well-validated sequence reference set is recommended over BLAST analysis using GenBank, which lacks quality control. In cases where treatment is species- or complex-dependent, clinicians should be made aware of the limitations of the technology used whenever results are reported, especially when closely related species are involved. The use of real-time PCR assays developed for specific complexes or species could speed up typing and facilitate interpretation of results, but currently no globally applicable methods are available. As previously recommended [15] and also apparent from this analysis, *hsp70* and the mini-exon currently offer the best *Leishmania* typing tools world-wide, and the use of ITS1 should be restricted to the Old World. Setting up similar evaluations outside Europe, in institutes in endemic as well as non-endemic countries, would shed additional light on the quality of *Leishmania* typing across the globe.

## References

[1] Alvar J Vélez ID Bern C Herrero M Desjeux P Cano J WHO Leishmaniasis Control Team. Leishmaniasis worldwide and global estimates of its incidence. PLoS One. 2012;7(5):e35671. 10.1371/journal.pone.0035671 22693548 PMC3365071

[2] World Health Organization. Control of the leishmaniasis: report of a meeting of the WHO Expert Committee on the Control of Leishmaniases, Geneva, 22-26 March 2010. Geneva, Switzerland: World Health Organization; 2010.

[3] Boelaert M, Sundar S. Leishmaniasis. In: Farrar J, Hotez P, Junghanss T, Kang G, Lalloo D, White N, eds. Manson's Tropical Diseases. 23 ed. Philadelphia: Elsevier Saunders, 2013. p. 631-51.

[4] Blum J Buffet P Visser L Harms G Bailey MS Caumes E LeishMan recommendations for treatment of cutaneous and mucosal leishmaniasis in travelers, 2014. J Travel Med. 2014;21(2):116-29. 10.1111/jtm.12089 24745041

[5] Blum J Lockwood DN Visser L Harms G Bailey MS Caumes E Local or systemic treatment for New World cutaneous leishmaniasis? Re-evaluating the evidence for the risk of mucosal leishmaniasis. Int Health. 2012;4(3):153-63. 10.1016/j.inhe.2012.06.004 24029394

[6] Bailey MS Green AD Ellis CJ O’Dempsey TJ Beeching NJ Lockwood DN Clinical guidelines for the management of cutaneous leishmaniasis in British military personnel. J R Army Med Corps. 2005;151(2):73-80. 10.1136/jramc-151-02-03 16097110

[7] Lawn SD Whetham J Chiodini PL Kanagalingam J Watson J Behrens RH New world mucosal and cutaneous leishmaniasis: an emerging health problem among British travellers. QJM. 2004;97(12):781-8. 10.1093/qjmed/hch127 15569809

[8] Akhoundi M Kuhls K Cannet A Votýpka J Marty P Delaunay P A historical overview of the classification, evolution, and dispersion of Leishmania parasites and sandflies. PLoS Negl Trop Dis. 2016;10(3):e0004349. 10.1371/journal.pntd.0004349 26937644 PMC4777430

[9] Van der Auwera G Dujardin JC. Species typing in dermal leishmaniasis. Clin Microbiol Rev. 2015;28(2):265-94. 10.1128/CMR.00104-14 25672782 PMC4402951

[10] Rioux JA Lanotte G Serres E Pratlong F Bastien P Perieres J. Taxonomy of Leishmania. Use of isoenzymes. Suggestions for a new classification. Ann Parasitol Hum Comp. 1990;65(3):111-25. 10.1051/parasite/1990653111 2080829

[11] El Baidouri F Diancourt L Berry V Chevenet F Pratlong F Marty P Genetic structure and evolution of the Leishmania genus in Africa and Eurasia: what does MLSA tell us. PLoS Negl Trop Dis. 2013;7(6):e2255. 10.1371/journal.pntd.0002255 23785530 PMC3681676

[12] Odiwuor S Veland N Maes I Arévalo J Dujardin JC Van der Auwera G. Evolution of the Leishmania braziliensis species complex from amplified fragment length polymorphisms, and clinical implications. Infect Genet Evol. 2012;12(8):1994-2002. 10.1016/j.meegid.2012.03.028 22516226

[13] Schönian G Nasereddin A Dinse N Schweynoch C Schallig HD Presber W PCR diagnosis and characterization of Leishmania in local and imported clinical samples. Diagn Microbiol Infect Dis. 2003;47(1):349-58. 10.1016/S0732-8893(03)00093-2 12967749

[14] Mauricio IL Stothard JR Miles MA. Leishmania donovani complex: genotyping with the ribosomal internal transcribed spacer and the mini-exon. Parasitology. 2004;128(Pt 3):263-7. 10.1017/S0031182003004578 15074875

[15] Van der Auwera G Ravel C Verweij JJ Bart A Schönian G Felger I. Evaluation of four single-locus markers for Leishmania species discrimination by sequencing. J Clin Microbiol. 2014;52(4):1098-104. 10.1128/JCM.02936-13 24452158 PMC3993476

[16] Van der Auwera G Maes I De Doncker S Ravel C Cnops L Van Esbroeck M Heat-shock protein 70 gene sequencing for Leishmania species typing in European tropical infectious disease clinics. Euro Surveill. 2013;18(30):20543. 10.2807/1560-7917.ES2013.18.30.20543 23929181

[17] Montalvo AM Fraga J Maes I Dujardin JC Van der Auwera G. Three new sensitive and specific heat-shock protein 70 PCRs for global Leishmania species identification. Eur J Clin Microbiol Infect Dis. 2012;31(7):1453-61. 10.1007/s10096-011-1463-z 22083340

[18] Fraga J Montalvo AM Maes L Dujardin JC Van der Auwera G. HindII and SduI digests of heat-shock protein 70 PCR for Leishmania typing. Diagn Microbiol Infect Dis. 2013;77(3):245-7. 10.1016/j.diagmicrobio.2013.07.023 24050933

[19] Marfurt J Nasereddin A Niederwieser I Jaffe CL Beck HP Felger I. Identification and differentiation of Leishmania species in clinical samples by PCR amplification of the miniexon sequence and subsequent restriction fragment length polymorphism analysis. J Clin Microbiol. 2003;41(7):3147-53. 10.1128/JCM.41.7.3147-3153.2003 12843055 PMC165364

[20] Roelfsema JH Nozari N Herremans T Kortbeek LM Pinelli E. Evaluation and improvement of two PCR targets in molecular typing of clinical samples of Leishmania patients. Exp Parasitol. 2011;127(1):36-41. 10.1016/j.exppara.2010.06.024 20599989

[21] Marfurt J Niederwieser I Makia ND Beck HP Felger I. Diagnostic genotyping of Old and New World Leishmania species by PCR-RFLP. Diagn Microbiol Infect Dis. 2003;46(2):115-24. 10.1016/S0732-8893(03)00040-3 12812715

[22] Bart A van Thiel PP de Vries HJ Hodiamont CJ Van Gool T. Imported leishmaniasis in the Netherlands from 2005 to 2012: epidemiology, diagnostic techniques and sequence-based species typing from 195 patients. Euro Surveill. 2013;18(30):20544. 10.2807/1560-7917.ES2013.18.30.20544 23929178

[23] Cortes S Rolão N Ramada J Campino L. PCR as a rapid and sensitive tool in the diagnosis of human and canine leishmaniasis using Leishmania donovani s.l.-specific kinetoplastid primers. Trans R Soc Trop Med Hyg. 2004;98(1):12-7. 10.1016/S0035-9203(03)00002-6 14702834

[24] de Bruijn MH Barker DC. Diagnosis of New World leishmaniasis: specific detection of species of the Leishmania braziliensis complex by amplification of kinetoplast DNA. Acta Trop. 1992;52(1):45-58. 10.1016/0001-706X(92)90006-J 1359760

[25] Noyes HA Reyburn H Bailey JW Smith D. A nested-PCR-based schizodeme method for identifying Leishmania kinetoplast minicircle classes directly from clinical samples and its application to the study of the epidemiology of Leishmania tropica in Pakistan. J Clin Microbiol. 1998;36(10):2877-81. 9738037 10.1128/jcm.36.10.2877-2881.1998 PMC105081

[26] Minodier P Piarroux R Gambarelli F Joblet C Dumon H. Rapid identification of causative species in patients with Old World leishmaniasis. J Clin Microbiol. 1997;35(10):2551-5. 9316906 10.1128/jcm.35.10.2551-2555.1997 PMC230009

[27] Schönian G Mauricio I Cupolillo E. Is it time to revise the nomenclature of Leishmania? Trends Parasitol. 2010;26(10):466-9. 10.1016/j.pt.2010.06.013 20609626

[28] Tamura K Peterson D Peterson N Stecher G Nei M Kumar S. MEGA5: molecular evolutionary genetics analysis using maximum likelihood, evolutionary distance, and maximum parsimony methods. Mol Biol Evol. 2011;28(10):2731-9. 10.1093/molbev/msr121 21546353 PMC3203626

[29] Institute of Tropical Medicine (ITG). *Leishmania* Typing Results, 2014. Table S1. Antwerp: ITG. [Accessed 29 Nov 2016]. Available from: www.itg.be/leishmaniatyping

[30] Zhang CY Lu XJ Du XQ Jian J Shu L Ma Y. Phylogenetic and evolutionary analysis of Chinese Leishmania isolates based on multilocus sequence typing. PLoS One. 2013;8(4):e63124. 10.1371/journal.pone.0063124 23646184 PMC3639960

[31] Piarroux R Fontes M Perasso R Gambarelli F Joblet C Dumon H Phylogenetic relationships between Old World Leishmania strains revealed by analysis of a repetitive DNA sequence. Mol Biochem Parasitol. 1995;73(1-2):249-52. 10.1016/0166-6851(95)00097-K 8577334

[32] Desbois N Pratlong F Quist D Dedet JP. Leishmania (Leishmania) martiniquensis n. sp. (Kinetoplastida: Trypanosomatidae), description of the parasite responsible for cutaneous leishmaniasis in Martinique Island (French West Indies). Parasite. 2014;21:12. 10.1051/parasite/2014011 24626346 PMC3952653

[33] Shaw J Pratlong F Floeter-Winter L Ishikawa E El Baidouri F Ravel C Characterization of Leishmania (Leishmania) waltoni n.sp. (Kinetoplastida: Trypanosomatidae), the Parasite Responsible for Diffuse Cutaneous Leishmaniasis in the Dominican Republic. Am J Trop Med Hyg. 2015;93(3):552-8. 10.4269/ajtmh.14-0774 26149864 PMC4559697

